# A systematic review of the incidence, management and prognosis of new-onset autoimmune connective tissue diseases after COVID-19

**DOI:** 10.1007/s00296-023-05283-9

**Published:** 2023-02-14

**Authors:** Koushan Kouranloo, Mrinalini Dey, Helen Elwell, Arvind Nune

**Affiliations:** 1 School of Medicine, University of Liverpool, Ashon St., Liverpool, L69 3GE UK; 2 Royal Liverpool University NHS Foundation Trust, Prescot St., Liverpool, L7 8XP UK; 3 Department of Rheumatology, Queen Elizabeth Hospital, Stadium Rd., London, SE18 4QH UK; 4 Institute of Life Health Sciences, University of Liverpool, Liverpool, L7 8TX UK; 5 BMA Library, BMA House, Tavistock Square, British Medical Association, London, WC1H 9JP UK; 6 Department of Rheumatology, Southport and Ormskirk NHS Foundation Trust, Southport, PR8 6PN UK

**Keywords:** COVID-19, SARS-CoV-2, Pandemic, Rheumatic disease, Autoimmune connective tissue diseases

## Abstract

A literature review on new-onset autoimmune connective tissue diseases (ACTDs) following COVID-19 is lacking. We evaluated potential associations between COVID-19 and the development of new-onset ACTDs. The “population” was adults with disease terms for ACTDs, including systemic lupus erythematosus (SLE), Sjogren’s syndrome, systemic sclerosis (SSc), idiopathic inflammatory myositis (IIM), anti-synthetase syndrome, mixed CTD and undifferentiated CTD, and “intervention” as COVID-19 and related terms. Databases were searched for English-language articles published until September 2022. We identified 2236 articles with 28 ultimately included. Of the 28 included patients, 64.3% were female, with a mean age was 51.1 years. The USA reported the most cases (9/28). ACTD diagnoses comprised: 11 (39.3%) IIM (including four dermatomyositis); 7 (25%) SLE; four (14.3%) anti-synthetase syndrome; four (14.3%) SSc; two (7.1%) other ACTD (one lupus/MCTD overlap). Of eight, four (14.3%) patients (including that with lupus/MCTD) had lupus nephritis. The average time from COVID-19 to ACTD diagnosis was 23.7 days. A third of patients were admitted to critical care, one for treatment of haemophagocytic lymphohistiocytosis in SLE (14 sessions of plasmapheresis, rituximab and intravenous corticosteroids) and nine due to COVID-19. 80% of patients went into remission of ACTD following treatment, while three (10%) patients died—one due to macrophage activation syndrome with anti-synthetase syndrome and two from unreported causes. Our results suggest a potential association between COVID-19 and new-onset ACTDs, notably in young females, reflecting more comprehensive CTD epidemiology. The most common diagnosis in our cohort was IIM. The aetiology and mechanisms by which ACTDs emerge following COVID-19 remain unknown and require further research.

**Supplementary Information:**

The online version contains supplementary material available at 10.1007/s00296-023-05283-9.

## Introduction

The COVID-19 pandemic resulted in substantial mortality and morbidity [1]. Globally, an estimated 6.49 million people have died due to COVID-19 and its complications [2]. Although primarily a respiratory disease, SARS-CoV-2 infection has been linked to hyperinflammation in multiple organs due to cytokine storm and molecular mimicry [3, 4]. Several new autoimmune and autoinflammatory conditions have been reported among the SARS-CoV-2 survivors [5–7]. A systematic review (SR) by Saad et al. discovered that SARS-CoV-2 infection is associated with neurological, cardiological, and musculoskeletal inflammatory diseases [8].

Similarly, recent studies have linked SARS-CoV-2 infection to the onset of systemic autoimmune rheumatic diseases (SARD) following SARS-CoV-2 infection [9, 10]. An SR by Chaudhry et al. elucidated that eight patients developed new rheumatoid arthritis (RA) and several others had flare-ups of their existing RA after being infected with SARS-CoV-2 [11], aligning with another SR of literature on the vasculitides after COVID-19 infection [12]. Despite the emergence of new-onset autoimmune connective tissue diseases (ACTDs) following COVID-19 infection, an SR of the literature is lacking. Our objectives were twofold: (i) to investigate the prevalence, clinical outcomes, treatment, and prognosis of new-onset ACTDs after SARS-CoV-2 infection and (ii) to evaluate the potential association between COVID-19 infection and the development of new-onset ACTDs in adults.

## Methods

This SR was conducted in accordance with the Cochrane Handbook and reported as per the Preferred Reporting Items for Systematic Reviews and Meta-Analyses [13, 14].

The protocol was developed and registered in the PROSPERO database of SRs (CRD42022358750). The review question was: Is there an association between COVID-19 infection and the development of new-onset ACTDs in adults? We assessed the incidence of new cases of ACTDs developing after COVID-19 infection and their clinical characteristics, treatment, and outcomes.

### Population

We included adults with ACTDs, including (but not limited to) systemic lupus erythematosus (SLE), Sjogren’s syndrome, systemic sclerosis (SSc), any idiopathic inflammatory myositis (IIM), anti-synthetase syndrome, mixed CTD and undifferentiated CTD (and related database specific indexing terms), with “intervention” as COVID-19 and related terms. All indexing terms and related keywords used are detailed within the supplementary materials.

We excluded patients developing new-onset ACTDs without prior SARS CoV-2 infection or patients without developing new-onset ACTD or flare of existing ACTDs.

Patients developing a systemic autoimmune rheumatic disease, not included in the above list, were excluded (such as inflammatory arthropathies and vasculitides).

### Outcome

Outcomes were demographics, clinical characteristics and disease trajectory, treatment, and timing of developing new-onset ACTDs after SARS-CoV-2.

Intervention and comparator descriptors were not applied to this review.

### Search strategy, databases and study selection

**Fig. 1:**
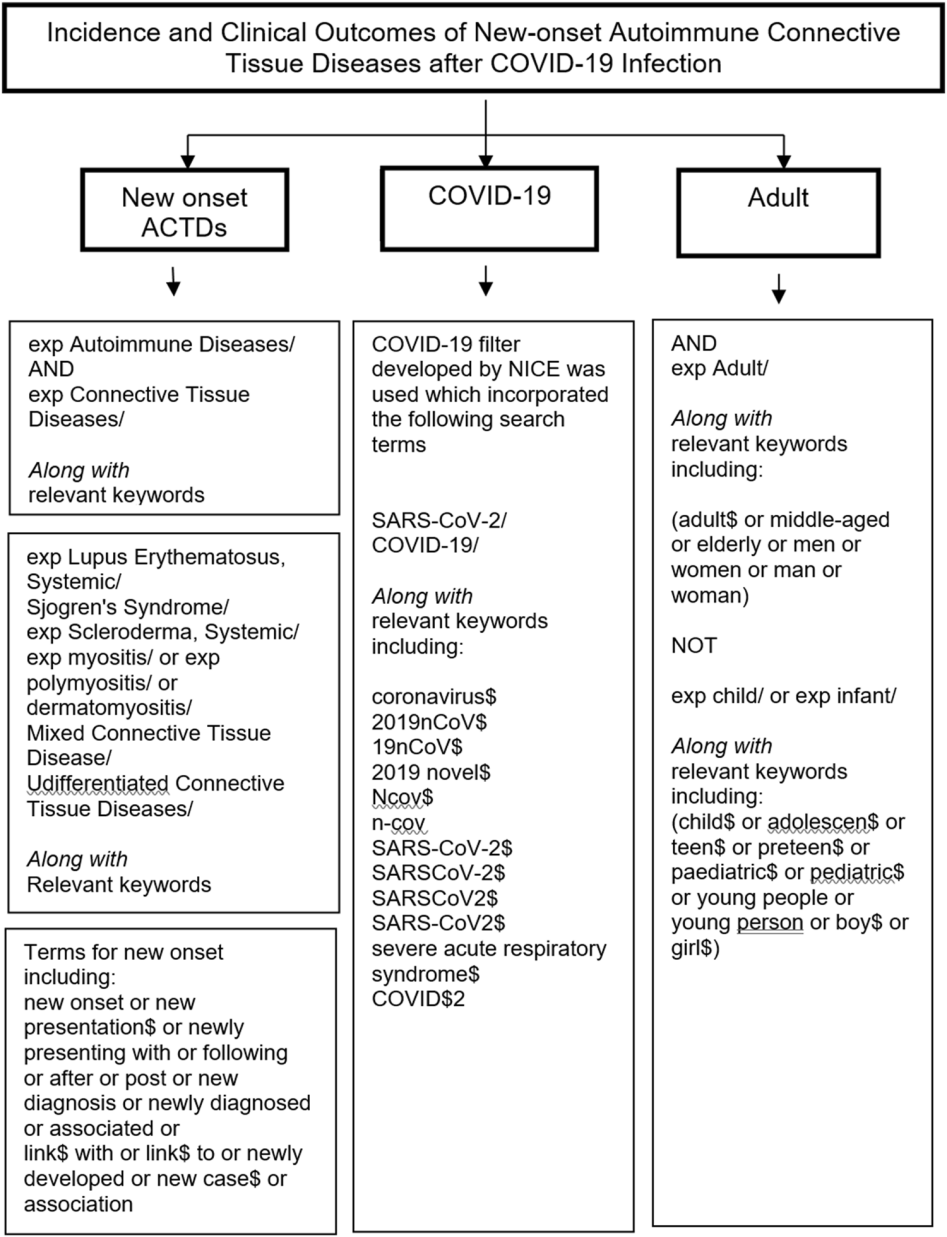
Flow chart of the search strategy

The search strategy is strategies are available in the online supplementary material. To ensure full comprehensive coverage, indexing terms (MeSH, applicable to Medline and Cochrane, and Emtree headings used on Embase) along with relevant keyword searching were incorporated. For terms for COVID-19, a dedicated search strategy developed by the National Institute for Clinical Excellence was used (Fig. 1). Medline, Embase, and Cochrane databases were searched from 2019 till September 2022, restricted to English-language articles only concerning adult populations. Eligible articles were: case reports and series (of any sample size), observational studies, qualitative studies and randomised controlled trials. Patients developing ACTDs without prior COVID-19 or reporting flares of existing ACTDs were excluded. Information was extracted on patient demographics, new ACTDs’ onset time, clinical characteristics, COVID-19 and ACTD treatment, and COVID-19 and ACTD outcomes.

**Fig. 2:**
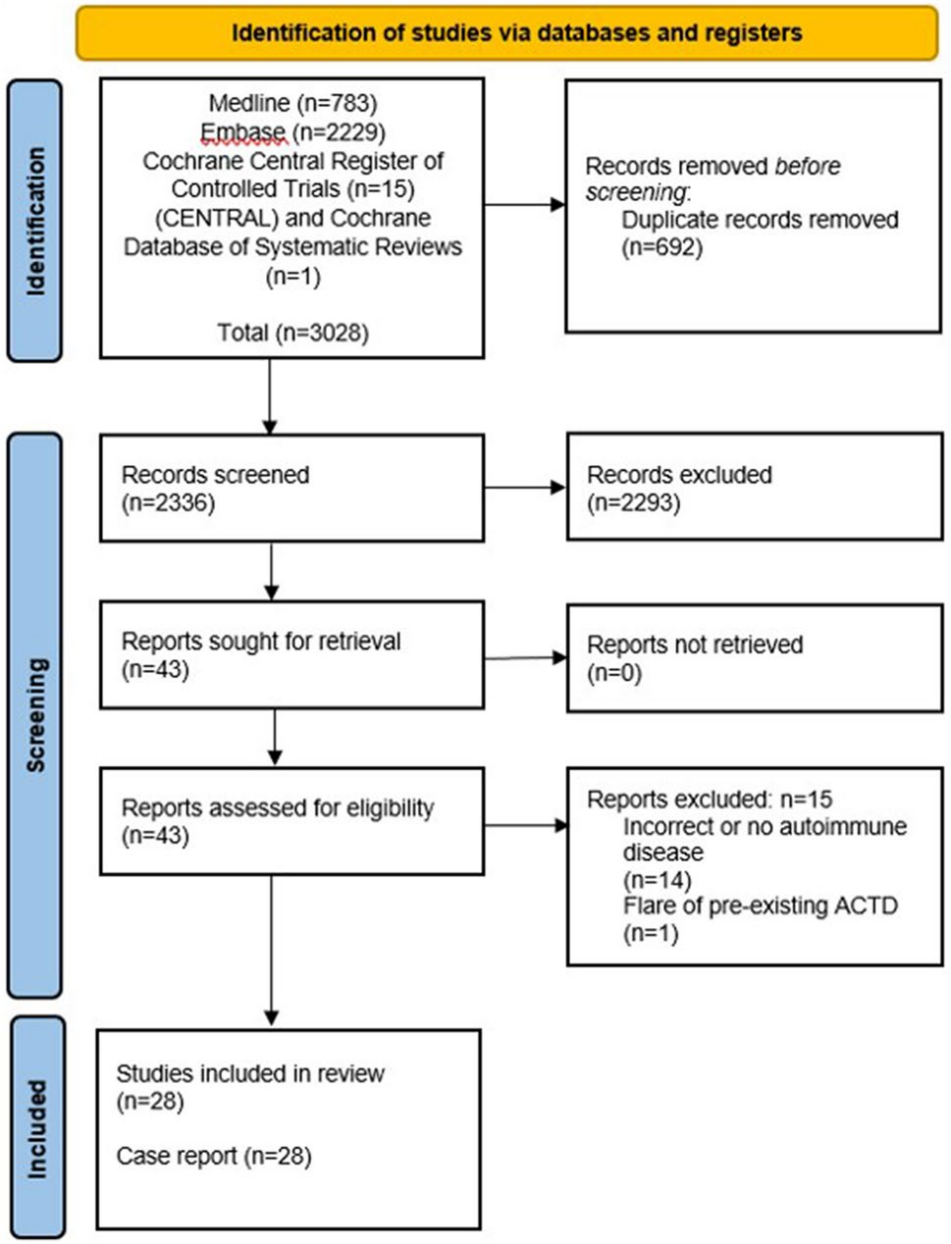
Flow diagram of stages of systematic literature review

Full-length articles were uploaded into EndNote V.X9 (Clarivate Analytics, Pennsylvania, USA), with duplicates removed (Fig. 2). Titles and abstracts were screened for eligibility, and articles meeting inclusion criteria were examined in further detail. For validation, 20% of the articles were screened. There were nil disagreements.

All retrieved articles were either case reports or case series; therefore, no formal risk of bias assessment was possible.

## Results

After deduplication, 2336 articles were identified. After screening the title and abstract, 2293 papers were excluded, with 43 proceeding to full-text screening. Ultimately, 28 articles (all single case reports) were included.

Article information and basic demographics are detailed in Table 1. Of the 28 included patients, 64.3% were female. The mean age was 51.1 years (range 20–89 years). Most case reports were from the USA (9/28), followed by Iran (4/28).

**Table 1:** Summary of included case reports, with basic demographics, comorbidities and final ACTD diagnosis

Title	Country	Gender	Mean age (years)	Comorbidities	ACTD diagnosis (as reported in article)
Zhang et al. 2022 [22]	USA	F	58	No	COVID‐19-associated myositis
Ramachandran et al. 2022 [30]	USA	M	53	Hypertension, CKD 3, cholecystectomy	SLE
Pereira et al. 2022 [40]	India	M	57	Unknown	Anti-synthetase syndrome
Okayasu et al. 2022 [23]	Japan	M	89	Hypertension, angina pectoris, dementia, clavicle fracture, previous lumbar vertebral compression fracture	Myositis and febrile neutropenia
Okada et al. 2022 [24]	Japan	F	64	Unknown	Dermatomyositis
Nunes et al. 2022 [38]	Portugal	F	70	Hypertension	Toxic epidermal necrolysis-like subacute cutaneous lupus
Kazzi et al. 2022 [31]	USA	M	37	No	SLE
Holzer et al. 2022 [19]	Germany	F	20	Unknown	Dermatomyositis
Giuggioli et al. 2022 [41]	Italy	F	53	Unknown	Raynaud's and systemic sclerosis
Chandra & Kahaleh 2022 [42]	USA	F	48	Anxiety, depression	Systemic Sclerosis
Bouchard Marmen et al. 2022 [43]	Canada	M	62	Unknown	Anti-synthetase syndrome
Blum et al. 2022 [44]	USA	M	67	None	Diffuse systemic sclerosis
Assar et al. 2022 [32]	Iran	F	38	Anxiety	SLE
Anderle et al. 2022 [45]	Austria	M	20	Unknown	Anti-MDA5 dermatomyositis
Amin et al. 2022 [27]	Pakistan	F	52	None	Polymyositis
Ali et al. 2022 [33]	Pakistan	F	22	Unknown	MCTD associated with a flare of lupus nephritis
Zamani et al. 2021 [34]	Iran	M	39	None	SLE
Slimani et al. 2021 [35]	Morocco	F	23	None	SLE and aPLS
Dadras et al. 2021 [25]	Iran	F	58	Diabetes mellitus, hypothyroidism, coronary artery	Dermatomyositis
Lokineni et al. 2021 [29]	USA	M	51	None	Necrotizing Myositis
Keshtkarjahromi et al. 2021 [46]	USA	F	65	Psoriasis, hypertension, hyperlipidaemia	MDA5-positive dermatomyositis complicated by MAS
Fineschi S 2021 [47]	Sweden	M	47	Unknown	Systemic Sclerosis
Borges et al. 2021 [20]	Brazil	F	36	Unknown	Dermatomyositis
Assar et al. 2021 [26]	Iran	F	45	OCD, hypothyroidism, migraine	Neutrophilic myositis
Ali et al. 2021 [36]	USA	F	25	Asthma, depression	SLE (complicated by HLH)
Aldaghlawi et al. 2021 [28]	USA	F	69	Stage IV chronic lymphocytic leukaemia	Myositis
Sacchi et al. 2020 [21]	Italy	F	77	Obesity, monoclonal gammopathy, diabetes mellitus, COPD, atrial fibrillation, CKD, cardiac failure	Myositis
Bonometti et al. 2020 [37]	Italy	F	85	None	SLE

*CKD* chronic kidney disease, *SLE* systemic lupus erythematosus, *MDA5* melanoma differentiation-associated protein 5, *COPD* chronic obstructive pulmonary disease

ACTD diagnoses comprised: 11 (39.3%) IIM (including 4 cases of dermatomyositis); 7 (25%) SLE; 4 (14.3%) anti-synthetase syndrome; 4 (14.3%) SSc; 2 (7.1%) other ACTD (one diagnosed with lupus/MCTD overlap). Of the eight patients diagnosed with SLE or lupus/MCTD, four (14.3%) were diagnosed with lupus nephritis. The average onset time from COVID-19 infection to ACTD diagnosis was 23.7 days.

The majority of cases (*n* = 16) were reported in 2022 and only one case of lupus nephritis was reported in 2020.

Investigations carried out varied markedly depending on geographic region (Table 2). Serum inflammatory markers (ESR and CRP) were gathered before the diagnosis of CTD in (9 ESR) and (14 CRP) cases with a mean of 70.2 mm/hr and 74.3 mg/L, respectively. One case had a normal ESR prior to diagnosis of ACTD and two patients had CRP levels reported within the normal range prior to ACTD diagnosis. Post-diagnosis of ACTD, there was reduction in both the ESR and CRP levels of those reported initially, with a mean of 53 mm/hr and 12.2 mg/L, respectively. One case had ESR within the normal range, and one had CRP within the normal range post-CTD diagnosis.

**Table 2:** Summary of investigations leading to ACTD diagnosis

Title	Time between onset of COVID-19 and autoimmune symptoms (days)	ESR pre-CTD-diagnosis (mm/hr)	CRP pre-CTD diagnosis (mg/L)	ESR post-CTD diagnosis and treatment (mm/hr)	CRP post-CTD diagnosis and treatment (mg/L)	CTD serology and basic biochemistry/haematology markers (where reported)	Imaging and findings (as reported)	Other investigations
Zhang et al. 2022 [22]	21	94	110	–	–	Positive anti-Ku, anti–SAE 1 IgG, anti–SS‐A	MRI: diffuse muscle oedema and enhancement, with region of myonecrosis	
Ramachandran et al. 2022 [30]	–	–	–	–	–	Positive ANA 1:1280, speckled pattern; dsDNA 150 IU/ml Low C3 and C4	Nil described	Renal biopsy: focal segmental glomerulosclerosis, collapsing variant. Light microscopy: mild podocyte hyperplasia, increase in mesangial cellularity and matrix. Severe interstitial fibrosis and tubular atrophy involving 70–80% of the cortical parenchyma with focal dense inflammation. Electron microscopy: glomeruli with global sclerosis and intracapillary deposits. Stage IV lupus nephritis
Pereira et al. 2022 [40]	56	–	–	–	–	Raised CPK (3736 IU/L) ANA and anti-smooth muscle antibodies were negative Positive anti-Jo-1	PET/CT MIP: abnormal increased FDG uptake in multiple muscle regions, more intense in the upper-limb muscles, suggesting possibility of inflammatory polymyositis (PM) and changes of interstitial lung disease with septal thickening and bronchiectatic changes in the right lower lobe	
Okayasu et al. 2022 [23]	28	–	117	–	–	ANA < 40 C3 126, C4 31 KL-6 332 MPO-ANCA < 1.0; Positive anti-SSA/Ro < 1.0; Anti-SSB/La autoantibodies < 1.0; CCP Antibodies < 0.6; Cardiolipin antibodies < 4.0; IgG4 111	MRI (STIR) thighs: irregular high-intensity areas in both adductor muscle groups, suggesting necrotizing fasciitis. CT chest: no obvious interstitial pneumonia or lymphadenopathy suspicious for lymphoid species	
Okada et al. 2022 [24]	28	–	–	–	–	Creatine kinase 1495 U/l D-dimer 6.1 µg/ml. Positive anti-NXP2	CT thorax, abdomen, pelvis: no malignancy nor interstitial lung disease MRI (STIR): intramuscular hyperintensity in proximal limbs	
Nunes et al. 2022 [38]	29	51	201	–	–	C3 0.66 g/L (0.83–1.93 g/L), C4 0.1 (0.15–0.57) Positive ANA 1:1,280, nuclear homogeneous pattern ENA antibodies SSA60 (Ro60)/SSB (La) positive	Nil described	Urine analysis: 24 h proteinuria: 642 mg/24 h (50–80 mg/24 h)
Kazzi et al. 2022 [31]	42	–	–	–	–	Positive ANA, positive double-stranded DNA antibody Hypocomplementemia, leukopenia	CT thorax, abdomen, pelvis: subtle bilateral infiltrates may be secondary to atypical pneumonia. Mild thoracic, abdominal, and pelvic lymphadenopathy, non-specific. Mild mesenteric stranding may be secondary to mesenteric panniculitis, with possible pancreatitis	Proteinuria
Holzer et al. 2022 [19]	14	–	< 5	–	–	CK 19,647 Positive ANA: 1:640 Positive anti-NXP2	MRI muscle: Bilateral myositis of muscles of the pelvic hip girdle and thighs	
Giuggioli et al. 2022 [41]	28	–	65	–	–	Positive ANA, anticentromere pattern	Nailfold capillaroscopy: “early scleroderma pattern”	
Chandra and Kahaleh 2022 [42]		–	–	–	–	Positive ANA > 1:1280	HRCT chest: interstitial lung disease, findings suggestive of non-specific interstitial pneumonia	
Bouchard Marmen et al. 2022 [43]	28	–	61	–	–	CK 7696, Positive ANA and anti-Jo-1	CT thorax: missed opacities with sub segmental consolidation MRI: oedema of the gluteal and thigh muscles consistent with myositis	
Blum et al. 2022 [44]	91	–	–	–	–	Positive ANA Positive anti-RNP and ani-SSA antibodies	CT chest: ground-glass opacities possibility indicating interstitial lung disease	
Assar et al. 2022 [32]	18	53	–	53	–	Positive ANA Positive anti-dsDNA Positive P-ANCA	CT thorax and abdomen: pericardial and pleural effusion and enlarged liver and abdominal lymph nodes	
Anderle et al. 2022 [45]	–	–	60	–	–	CK 246 U/L Positive ANA, fine speckled pattern 1:320 Anti-Ro-60 Ab at 23 U/mL (ULN ≤ 10 U/mL) Aanti-MDA-5 14 U/mL (ULN ≤ 10 U/mL)	CT chest (week 2): patchy ill-defined consolidations and areas of ground-glass opacifications in the periphery of both lower lobes and subtle thickening of the bronchial walls and hepatic steatosis 3-Tesla, gadolinium contrast enhanced MRI: T2 fat saturated bilateral hyperintense signal alterations of bilateral proximal thigh muscles compatible with myositis	
Amin et al. 2022 [27]	112	–	40.5	–	12.2	Nil described	CT thorax, abdomen, pelvis: enlarged fatty liver and atrophic left kidney MRI shoulder and hip muscles: inflammatory changes in the muscles of the shoulder and pelvic girdle, chest, and anteromedial and lateral compartments of the thigh	
Ali et al. 2022 [33]	–	102	153	–	–	Positive ANA, anti-smith (Sm) and U1 small nuclear ribonucleoprotein (U1-RNP) Positive rheumatoid factor C3 and C4 within range	Nil described	
Zamani et al. 2021 [34]	56	74	34	Normal	Normal	Total complement activity (CH50), 45 (50–150); C3 133 mg/dL (90–180 mg/dL); C4 14 mg/dL (10–40 mg/dL) Anti-La/SSB, 160 U/ml (< 12 U/mL); anti-SSA/Ro, 200 U/mL (< 25 U/mL) Anti-CCP 48 IU/mL (< 20 IU/mL) Anti-dsDNA 70 IU/mL (< 35 IU/mL) Positive fluorescence ANA 1/160. Anticardiolipin, lupus anticoagulant, anti-beta-2 glycoprotein 1, C-ANCA, P-ANCA were negative	CT chest: two ground-glass opacity nodules in the lower lobes of both lungs	Renal biopsy: mild mesangial hypercellularity (lupus nephritis class I)
Slimani et al. 2021 [35]	–	–	–	–	–	Elevated PT, APTT Positive ANA, ds-DNA, anti-cardiolipin, beta-2-glycoprotein, lupus anticoagulant	Nil described	
Dadras et al. 2021 [25]	–	57	–	–	–	ANA, anti‐ds‐DNA, anti‐Smith antibody negative Myositis‐specific antibodies including anti‐Mi‐2, ‐Ku, ‐PM/Scl‐100, ‐Scl‐75, ‐SRP, ‐PL‐7, ‐PL‐12, ‐EJ, ‐OJ, ‐Jo‐1, and ‐Ro‐52 were negative	CT abdomen and pelvis: normal CT chest: bilateral multifocal patchy consolidations with reverse halo view suggestive of the chronic phase of organising COVID‐19 pneumonia	Three skin biopsies from different skin sites were taken with differential diagnoses of dermatomyositis and lupus erythematosus; the first was sent for examination under direct immune fluorescence, with findings in favour of lupus erythematosus. The second (from a Gottron papule) and third (from a vesicle on the extremities) biopsies were evaluated using hematoxylin‐eosin staining; findings indicated dermatomyositis‐lupus overlap features and were compatible with a collagen vascular disease
Lokineni et al. 2021 [29]	84	–	–	–	–	Nil described	Unknown	
Keshtkarjahromi et al. 2021 [46]	56	20	67	–	–	Positive ANA, anti-MDA5, SSA-52 (Ro) Low C3	1^st^ admission: Diagnostic imaging included MRI of right femur that demonstrated multiple scattered areas of proximal muscle oedema, which while non-specific, was felt to be consistent with an inflammatory myositis CT chest: mild bilateral patchy infiltrates 2^nd^ admission: Repeat CT demonstrated a new, marked consolidative processes within the bilateral lower lobes in a peripheral distribution with pleural sparing	Skin biopsy of the anterior chest was subsequently performed which demonstrated vacuolar interface dermatitis with an increase in dermal mucin
Fineschi S 2021 [47]	21	Normal	Normal	–	–	Strongly positive ANA, nucleolar pattern Positive Anti-PM/Scl 75 and PM/Scl 100 Anti-Scl-70, anti-Jo-1, anti-RNA-polymerase III, and other autoantibodies tested negative	HCRT: ground-glass opacities with predominantly peripheral and subpleural distribution such as in the early stages of interstitial lung disease	
Borges et al. 2021 [20]	14	–	–	–	–	Positive fine speckled pattern ANA (1/640) Positive anti-Mi2, CPK 3518U/l		Skin biopsy showed lamellar keratosis with foci of vascular changes in the epidermal layer and dilated vessels with a thickened wall and perivascular lymphocytic infiltrate
Assar et al. 2021 [26]	112	87	–	–	–	Normal ANA, anti-dsDNA, antiphospholipid, anti-Ro, anti-La, ANCA, anti Jo1 antibodies	CT chest: peripheral and multi-lobar fibrotic areas in the lingula, right middle lobe and upper zones which were consistent with fibrotic changes due to previous COVID-19 infection	Electromyography and nerve conduction velocity studies (EMG/NCV) were compatible with inflammatory myopathy. There was no evidence of neuropathy and radiculopathy
Ali et al. 2021 [36]	14	–	Normal	–	–	Positive anti-dsDNA, anti-Smith, anti-RNP, anti-Ro, anti-La	Echocardiogram: large pericardial effusion	
Aldaghlawi et al. 2021 [28]	21	–	–	–	–	CPK 2713 µ/L, lactate dehydrogenase 1348 µ/L, haptoglobin 196 mg/dL, haemoglobin 11.7 gm/dL, platelets 75 k/mm3, aspartate aminotransferase 96 µ/L, alanine aminotransferase 72 µ/L, creatinine 0.6 mg/dL, prothrombin 12.3 s, partial thromboplastin time 32.5 s, fibrinogen 599 mg/dL, IGG 333 mg/dL, immunoglobulin M 26 mg/dL, immunoglobulin A 83 mg/dL Peripheral blood smear revealed marked agglutination of red blood cells and a cold agglutinin with thermal amplitude of 30 °C was identified with complement C3B and C4 identified on red blood cell	Unknown	Hepatitis B and C viral serologies were negative for acute infection
Sacchi et al. 2020 [21]		–	59.4	–	–	Positive ANA, cytoplasmic pattern (1:320) granular type, Anti-Ku and anti-MI 2b positivity	Unknown	
Bonometti et al. 2020 [37]	–	–	–	–	–	Positive ANA with cytoplasmic (1: 160), homogeneous (1: 320) and granular (1: 320) pattern, Ku positivity and atypical ANCA	Unknown	

*ESR* erythrocyte sedimentation rate, *CRP* C-reactive protein, *ANA* anti-nuclear antibody, *ANCA* antineutrophil cytoplasmic antibodies, *CT* computed tomography, *MRI* magnetic resonance imaging, *CTD* Connective tissue diseases, *DsDNA* double-stranded deoxyribonucleic acid, *CPK* creatinine phosphokinase, *COVID-19* Coronavirus disease 2019, *CT* computed tomography, *RNP* ribonucleoprotein

Regarding autoantibody levels (Table 2), anti-nuclear antibody (ANA) was the most commonly positive autoantibody in this cohort (*n* = 16), with a speckled pattern most commonly described. Where ANA was reported, two cases reported normal ANA titres and normal levels for the remaining autoantibody panel (including myositis-specific antigens). Details regarding all other autoantibodies are available in Table 2.

The most common imaging modality reported for our patients was computed tomography (CT) of the chest (*n* = 17) with the most common finding being “changes suggestive of interstitial lung disease (*n* = 13). Four cases did not find any pulmonary changes, out of which three cases had magnetic resonance (MRI) evidence suggestive of inflammatory myositis. Seven cases had MRI imaging of the muscles, which demonstrated muscle oedema suggestive of inflammatory myositis; one had electromyography to confirm the diagnosis.

The most commonly diagnosed CTD in our review was IIM, with 11 cases identified, four dermatomyositis. There was a wide age range (20–89 years), with female predominance (*n* = 9). CTD symptoms onset time also varied markedly, ranging from 14 to 112 days since COVID-19 diagnosis. Autoantibody serology also varied, with just three cases reporting positive ANA [19–21] and six reporting positivity for other autoantibodies, including NXP2, Mi2, Ku, and Ro [19–24]. In three cases with negative autoantibody serology, a diagnosis of myositis was made based on MRI muscle imaging, skin biopsy histology (consistent with dermatomyositis) and electromyography findings [23, 25–27]. Three cases did not report serology or imaging justification of diagnosis, with these diagnoses based on classic symptoms including “malaise, muscle weakness and skin lesions” and “severe intractable pain in bilateral lower extremities and subjective pelvic girdle weaknesses’’ associated with a high creatinine phosphokinase level [25, 28, 29].

COVID-19 treatment differed depending on the stage of pandemic and the country. Ten patients were admitted to critical care, one for ACTD treatment for SLE with haemophagocytic lymphohistiocytosis (HLH; 14 sessions of plasmapheresis, rituximab and intravenous corticosteroids) and nine for COVID-19. Five cases made explicit comments about the severity of COVID-19. However, no articles specified which grading system was used. There are several COVID-19 severity indices available, e.g. National Institute for Health, World Health Organisation, but none were mentioned in the texts. Nonetheless, three were classified as “mild”, one as “low severity” and one as “severe”.

Seventeen case reports provided details of treatment for COVID-19 (either the details of therapies given, or the fact that none were administered; Table 3). The following specific treatments were described for these patients: one case received a combination of tocilizumab, anticoagulation, hydroxychloroquine, and azithromycin; one received tapering corticosteroids and nintedanib for post-COVID-19 lung fibrosis; one received supplemental oxygen, dexamethasone, ipratropium bromide and enoxaparin; one received azithromycin, hydroxychloroquine; one received naproxen and Diphenhydramine syrup; one received hydroxychloroquine, cefazolin and azithromycin; one received “broad-spectrum antibiotics”, convalescent plasma and dexamethasone; one received remdesivir, corticosteroids, colchicine and plasmapheresis; one received levofloxacin and dexamethasone; one received oxygen, lopinavir/ritonavir, hydroxychloroquine, doxycycline, ceftriaxone and anticoagulant. Two cases of COVID-19 infection received no treatment.

**Table 3:** Summary of treatment and outcomes for COVID-19 and ACTD

Title	Severity of COVID-19	Treatment of COVID-19	ACTD diagnosis	Prior ACTD diagnosis	Treatment of CTD	ITU admission	CTD remission	Outcome
Zhang et al. 2022 [22]	Unknown	Tocilizumab, anticoagulation (drug name not specified), hydroxychloroquine, azithromycin	COVID‐19–associated myositis	No	IV methylprednisolone	No	Yes	Survived
Ramachandran et al. 2022 [30]	Unknown	Unknown	SLE	No	IV methylprednisolone 1 g/day for 3 days, then oral prednisolone 60 mg, with plasmapheresis (6 rounds), mycophenolate and hydroxychloroquine	No	Yes	Survived
Pereira et al. 2022 [40]	Unknown	Tapering corticosteroids and nintedanib for post-COVID lung fibrosis	Anti-synthetase syndrome	Unknown	Mycophenolate mofetil	No	Yes	Survived
Okayasu et al. 2022 [23]	Unknown	Unknown	Myositis and febrile neutropenia	No	Oral prednisolone 50 mg/day for 5 days	Unknown	Yes	Survived
Okada et al. 2022 [24]	Unknown	Unknown	Dermatomyositis	Unknown	1 g IV methylprednisolone for 3 days, then oral prednisolone 60 mg/day	No	Yes	Survived
Nunes et al. 2022 [38]	Unknown	Supplemental oxygen, dexamethasone 6 mg/day, ipratropium bromide 40 µg 6-hourly, enoxaparin 40 mg/day, paracetamol 1 g as required	Toxic epidermal necrolysis-like subacute cutaneous lupus	No	Continuous surveillance and balneotherapy for 10 days. Subsequent hydroxychloroquine 400 mg/day and prednisolone 1 mg/kg/day (dose not specified)	Yes	Yes	Survived
Kazzi et al. 2022 [31]	Low	None	SLE	No	Antibiotics, corticosteroids and MMF 1500 mg twice daily with resolution. Subsequent hydroxychloroquine (dose unspecified)	No	Yes	Survived
Holzer et al. 2022 [19]	Unknown	Unknown	Dermatomyositis	Unknown	Corticosteroids, IVIG, MMF, ciclosporin A, tofacitinib, rituximab	No	Yes	Survived
Giuggioli et al. 2022 [41]	Unknown	Azithromycin for 5 days; hydroxychloroquine 400 mg twice a day for 1 day and then 200 mg every 12 h for 5 days	Raynaud's and systemic sclerosis	No	Nifedipine for Raynaud’s	No	Yes	Survived
Chandra & Kahaleh 2022 [42]	Unknown	Unknown	Systemic Sclerosis	No	MMF 1500 mg twice daily, amlodipine 5 mg daily, methotrexate 12.5 mg once weekly, prednisone 5 mg twice daily	No	No	Survived
Bouchard Marmen et al. 2022 [43]	Unknown	Unknown	Anti-synthetase syndrome	Unknown	Pulsed IV methylprednisolone, then oral prednisolone, cyclophosphamide, rituximab, IVIG	Yes	Yes	Survived
Blum et al. 2022 [44]	Unknown but complicated by CCF, AF and PE post-COVID infection	Unknown	Diffuse systemic sclerosis	No	MMF	Yes	No	Died
Assar et al. 2022 [32]	Mild	Naproxen 500 mg twice daily and diphenhydramine syrup four times a day orally on outpatient basis	SLE	No	Prednisolone 30 mg daily, hydroxychloroquine 200 mg daily and azathioprine 150 mg daily followed by MMF	No	Yes	Survived
Anderle et al. 2022 [45]	Unknown	Unknown	Anti-MDA5 dermatomyositis		Corticosteroid pulsed therapy (250 mg intravenous prednisolone), acyclovir and trimethoprim/sulfamethoxazole, cyclophosphamide and tacrolimus due to rapid disease progression. Colchicine due to the hyperinflammatory state. Caspofungin, piperacillin/tazobactam and doxycycline administered for infection prophylaxis	Yes	Yes	Survived (required ECMO and double lung transplant)
Amin et al. 2022 [27]	Unknown	Unknown	Polymyositis	No	Oral prednisolone 60 mg/day, azathioprine 50 mg twice a day	No	Yes	Survived
Ali et al. 2022 [33]	Unknown	Unknown	MCTD associated with a flare of LN	Unknown	IV methylprednisolone 50 mg once daily throughout hospitalisation, in addition to oral hydroxychloroquine 200 mg once daily	No	Yes	Survived
Zamani et al. 2021 [34]	Mild	400 mg hydroxychloroquine twice on the first day and 200 mg twice daily for a further 6 days	SLE		Prednisolone 30 mg daily and hydroxychloroquine, gabapentin, and vitamin B (300 mg daily)	No	Yes	Survived
Slimani et al. 2021 [35]	Unknown	Unknown	SLE and aPLS	No	Nil described	Yes	No	Died
Dadras et al. 2021 [25]	Unknown	Cefazolin (2 g three times daily) and azithromycin (500 mg daily)	Dermatomyositis	Yes	Prednisolone 60 mg daily, methotrexate 15 mg weekly, hydroxychloroquine 400 mg daily	Unknown	Yes	Survived
Lokineni et al. 2021 [29]	Unknown	Broad-spectrum antibiotics (unspecified), convalescent plasma, dexamethasone	Necrotizing Myositis	No	Oral prednisone 60 mg daily, azathioprine 150 mg daily	No	Yes	Survived
Keshtkarjahromi et al. 2021 [46]	Unknown	Unknown	MDA5-positive dermatomyositis complicated by MAS	Yes	1^st^ admission: oral prednisone 60 mg daily, tapering regime. Discharged to rehabilitation centre with plans to continue steroid therapy with adjunctive trimethoprim-sulfamethoxazole for pneumocystis pneumonia prophylaxis 2^nd^ admission: IV methylprednisolone 1 g/day for 3 days followed by 80 mg IV daily, IVIG 400 mg/kg/ day for 5 days	Yes	No	Died
Fineschi S 2021 [47]	Mild	Unknown	Systemic Sclerosis	No	Calcium channel blocker, proton pump inhibitor, tear substitution	No	Unknown	Survived-awaiting further decision re immunosuppression
Borges et al. 2021 [20]	Unknown	Unknown	Dermatomyositis	Unknown	Pulsed IV methylprednisolone (unspecified) 5 days	No	Yes	Survived
Assar et al. 2021 [26]		Remdesivir, high doses of corticosteroids (unspecified), colchicine, plasmapheresis	Neutrophilic myositis	Yes	IVIG, 2 g/kg in four divided doses, prednisolone 1 mg/kg/day with gradual tapering (absolute doses unspecified)	Yes, for COVID No for CTD	Yes	Survived
Ali et al. 2021 [36]	Unknown	None	SLE (complicated by HLH)	No	MMF 250 mg daily, hydroxychloroquine 400 mg daily. 14 sessions of plasmapheresis, 600 mg of rituximab twice, high-dose corticosteroids (dose unspecified)	Yes, for CTD	Not specified	Survived- requiring long-term rehabilitation
Aldaghlawi et al. 2021 [28]	Severe	Levofloxacin 750 mg daily, dexamethasone 6 mg daily. Discharged on supplemental oxygen 2 l on day 16	Myositis	No	Oral prednisone 1 mg/kg daily for 4 weeks (dose unspecified); rituximab 375 mg/m2 weekly × 4 doses was initiated for cold agglutinin haemolytic anaemia; IVIG 1 g/kg daily × 2 doses on day 21 to address possible immune-related thrombocytopenia	No	Yes	Survived
Sacchi et al. 2020 [21]	Unknown	Oxygen supplementation then continued positive airway pressure therapy. Lopinavir/ritonavir, hydroxychloroquine, doxycycline, ceftriaxone, anticoagulation (unspecified)	Myositis	No	Corticosteroid 1 mg/kg (dose and type unspecified)	Yes, for COVID No for CTD	Yes	Survived
Bonometti et al. 2020 [37]	Swab negative, immunoglobulin positive	Unknown	SLE	No	Hydroxychloroquine and high-dose corticosteroids	No	Yes	Survived

Regarding CTD treatment, of those described (27), different strengths of corticosteroids (methylprednisolone in 8 and oral prednisone in 15) were the most frequently prescribed (Table 3). This was followed by hydroxychloroquine (*n* = 9), mycophenolate mofetil (MMF) (*n* = 8), rituximab (*n* = 4), intravenous immunoglobulins (*n* = 3), azathioprine (*n* = 3), methotrexate (*n* = 2), cyclophosphamide (*n* = 2), plasmapheresis (*n* = 1), ciclosporin (*n* = 1), tofacitinib (*n* = 1), nifedipine (*n* = 1), tacrolimus (*n* = 1) and colchicine (*n* = 1). Antibiotic prophylaxis was administered in two cases, and vitamins B and D each in one case. The majority (80%) of patients experienced remission of ACTD following treatment. In comparison, three (10%) patients died—one from macrophage activation syndrome associated with anti-synthetase syndrome and two from unknown causes.

## Discussion

This SR summarised the data on new-onset ACTDs following infection with SARS-CoV-2. Our findings from the 28 included cases suggest a potential association between COVID-19 infection and new-onset ACTDs, particularly in young females, reflective of wider CTD epidemiology. To our knowledge, this is the first SR to examine the association between COVID-19 and new-onset ACTDs, including the temporal relationship, diagnostic parameters and treatment.

Since March 2020, as the COVID-19 pandemic has progressed, so has our understanding of clinical sequelae arising following the infection. During the early stages of the pandemic, it was recognised that SARS-CoV-2 infection could cause a flare in SARD, including CTDs, which was well reported in the literature [15, 16]. ANA positivity was noted in 25% of hospitalised patients with acute COVID-19 infection, with a proportion of patients presenting with rheumatic manifestations, such as muscle weakness for myositis and rash and arthralgia for SLE, as in some of the cases described herein [16, 17]. An association was observed between severe COVID-19 and multisystem inflammatory syndromes and “cytokine storm”, similar to HLH and macrophage activation syndrome previously associated with ACTDs [17, 18]. Likewise, a temporal association between acute COVID-19 infection and the onset of ACTD became apparent in increasing number of cases.

The most common CTD following COVID-19 infection identified in our cohort was IIM, with four cases of dermatomyositis. Interestingly, IIM was diagnosed with negative autoantibody serology and solely on imaging or histology finding. In some cases, no investigative finding was reported, and a diagnosis was made based on symptoms [25, 28, 29]. It remains to be seen whether a subset of IIM is required within the nomenclature to account for such diagnoses arising post-COVID-19 infections, especially in the absence of typical serology.

Seven patients included in our review were diagnosed with SLE, and one with lupus/mixed connective tissue disease (MCTD) overlap [30–37]. Of these eight patients, four presented with lupus nephritis [30, 31, 33, 34]. Again, a wide age range was noted (22–85 years) with, 75% (6/8) of cases being female. One patient with SLE and antiphospholipid syndrome died following admission to the intensive therapy unit (ITU), although it was not specified which treatment she received for either COVID-19 or SLE [35]. Most patients required high-dose intravenous corticosteroids, followed by DMARDs, such as mycophenolate mofetil or hydroxychloroquine. One patient received plasmapheresis and rituximab after requiring ITU admission to treat ACTD and associated HLH [36]. In addition, Nunes et al. reported a case of toxic epidermal necrolysis-like lupus presentation following SARS-Cov-2 infection in a 70-year-old female with hypertension, who went into CTD remission following treatment with hydroxychloroquine and corticosteroids [38].

Our findings were consistent with those of Chaudry et al. [11], who conducted a similar literature review and discovered limited evidence of inflammatory arthritis developed following COVID-19 infection. However, this could be explained by the possible heterogeneity of the cases. On the other hand, an SR of case reports and case series by Wong et al. [12] found an association between COVID-19 and vasculitis. Moreover, COVID-19 has been linked to cytokine storm leading to an immune response to small vessel damage causing vasculitis and other immune-mediated inflammatory diseases [48]. Therefore, more research is needed to investigate the association between ACTDs and COVID-19.

The aetiology and mechanisms by which ACTDs emerge following COVID-19 infection remain unknown and require more robust epidemiological data. It is possible that patients had a mild asymptomatic disease in a genetically predisposed individual prior to COVID-19 infection, with SARS-CoV-2 triggering a flare due to the hyperinflammatory state [3]. Machado et al. recently proposed a new entity of “COVID-19-associated arthritis” in a similar review of inflammatory arthritis following COVID-19 infection. It may be that such nomenclature is required for those developing ACTD following SARS-COv-2 infection [39]. Further studies to elucidate the pathogenesis and aetiology of new-onset ACTD in these cases will aid the characterisation and understanding of these diseases.

### Strengths and limitations

Our SR included a small number of cases due to the specific area of rheumatology it covered and the rarity of the ACTD subset we investigated. This might have resulted in biased results, and it is important not to infer causality solely from these cases. However, it is an important subset of ACTDs following SARS-CoV-2 infection, which are relatively unexplored. Our findings will pave the way for future research and better care for ACTD patients. This SR only included case reports that were limited in establishing a cause–effect relationship and, thus, were not generalisable. Therefore, extensive and longitudinal studies to determine causation are recommended to supplement the current literature.

In conclusion, we summarised 28 cases of new-onset ACTD in this SR, the most common presentations being IIM and SLE. However, cases of SSc and rarer diseases such as anti-synthetase syndrome were also identified. Further epidemiological studies of ACTD diagnosed post-COVID-19 infection will help us better understand this association and help identify those at risk of developing ACTD after contracting SARS-CoV-2 infection.

## Supplementary Information

Below is the link to the electronic supplementary material.

Supplementary file1 (DOCX 21 KB)

Supplementary file2 (DOCX 17 KB)

## Data Availability

Data available upon request.
