# Supplementary material for: A systematic review of the incidence, management and prognosis of new-onset autoimmune connective tissue diseases after COVID-19
Source: Rheumatol Int. 2023 Feb 14;43(7):1221–43. doi: 10.1007/s00296-023-05283-9 (PMC9927056; doi:10.1007/s00296-023-05283-9)
Supplement: Supplementary file 2 — Supplementary file2 (DOCX 17 KB) [file 296_2023_5283_MOESM2_ESM.docx]

**Search strategies**

**A.1 Medline**

Ovid MEDLINE(R) ALL <1946 to September 01, 2022> Results per line

Date: 02/09/2022

**1** exp Autoimmune Diseases/ 527546

**2** exp Connective Tissue Diseases/ 330700

**3** 1 and 2 196323

**4** (autoimmune connective tissue disease$ or autoimmune connective

tissue disorder$ or systemic autoimmune disease$ or autoimmune

CTDs).ti,ab,kw,kf. 4027

**5** (ACTD or ACTDs).ti,ab,kw,kf. 365

**6** actinomycin D.ti,ab,kw,kf. 14087

**7** 5 not 6 76

**8** exp Lupus Erythematosus, Systemic/ 65126

**9** Sjogren's Syndrome/ 14132

**10** exp Scleroderma, Systemic/ 22481

**11** exp myositis/ or exp polymyositis/ or dermatomyositis/ 21836

**12** Mixed Connective Tissue Disease/ 1698

**13** Undifferentiated Connective Tissue Diseases/ 126

**14** (systemic lupus erythematosus or SLE or lupus or Sjo?gren$

syndrome or systemic scleroderma or systemic sclerosis or

inflammatory myopath$ or necrotizing autoimmune myopathy or

myositis or polymyositis or dermatomyositis or anti-synthetase

syndrome or antisynthetase syndrome or mixed connective tissue

disease or mixed connective tissue disorder or mixed collagen disease

or undifferentiated connective tissue disease or undifferentiated

connective tissue disorder or undifferentiated connective tissue

dysplasia or UCTD).ti,ab,kw,kf. 142016

**15** or/3-4,7-14 279606

**16** (new onset or new presentation$ or newly presenting with or following

or after or post or new diagnosis or newly diagnosed or associated or

link$ with or link$ to or newly developed or new case$ or

association).ti,ab,kw,kf. 11390419

**17** (earlier adj3 infect$).ti,ab,kw,kf. 1785

**18** or/16-17 11391045

**19** 15 and 18 115311

**20** SARS-CoV-2/ or COVID-19/ 185685

**21** (corona$ adj1 (virus$ or viral$)).ti,ab,kw,kf. 5437

**22** (CoV not (Coefficien$ or "co-efficien$" or covalent$ or Covington$ or

covariant$ or covarianc$ or "cut-off value$" or "cutoff value$" or "cutoff

volume$" or "cutoff volume$" or "combined optimi#ation value$" or

"central vessel trunk$" or CoVR or CoVS)).ti,ab,kw,kf. 99084

**23** (coronavirus$ or 2019nCoV$ or 19nCoV$ or "2019 novel$" or Ncov$ or

"n-cov" or "SARS-CoV-2$" or "SARSCoV-2$" or SARSCoV2$ or

"SARS-CoV2$" or "severe acute respiratory syndrome$" or

COVID$2).ti,ab,kw,kf. 299057

**24** or/20-23 306310

**25** 19 and 24 849

**26** exp Adult/ 7836719

**27** (adult$ or middle-aged or elderly or men or women or man or

woman).ti,ab,kw,kf. 3389650

**28** or/26-27 9198531

**29** 25 and 28 338

**30** exp child/ or exp infant/ 2707837

**31** (child$ or adolescen$ or teen$ or preteen$ or paediatric$ or pediatric$

or young people or young person or boy$ or girl$).ti,ab,kw,kf. 2112845

**32** or/30-31 3429939

**33** 25 not 32 783

**34** 29 or 33 805

**35** exp animals/ 25760922

**36** humans/ 20719209

**37** 35 not 36 5041713

**38** 34 not 37 801

**39** limit 38 to english language 783

**A.2 Embase**

Embase <1974 to 2022 August 31> Results per line

Date: 02/09/2022

**1** exp Autoimmune Diseases/ 665677

**2** exp Connective Tissue Diseases/ 489699

**3** 1 and 2 388682

**4** (autoimmune connective tissue disease$ or autoimmune connective

tissue disorder$ or systemic autoimmune disease$ or autoimmune

CTDs).ti,ab,kw,kf. 6443

**5** (ACTD or ACTDs).ti,ab,kw,kf. 450

**6** actinomycin D.ti,ab,kw,kf. 13958

**7** 5 not 6 126

**8** exp Lupus Erythematosus, Systemic/ 103059

**9** Sjogren's Syndrome/ 14837

**10** exp Scleroderma, Systemic/ 34363

**11** exp myositis/ or exp polymyositis/ or dermatomyositis/ 43583

**12** Mixed Connective Tissue Disease/ 4143

**13** Undifferentiated Connective Tissue Diseases/ 340

**14** (systemic lupus erythematosus or SLE or lupus or Sjo?gren$

syndrome or systemic scleroderma or systemic sclerosis or

inflammatory myopath$ or necrotizing autoimmune myopathy or

myositis or polymyositis or dermatomyositis or anti-synthetase

syndrome or antisynthetase syndrome or mixed connective tissue

disease or mixed connective tissue disorder or mixed collagen disease

or undifferentiated connective tissue disease or undifferentiated

connective tissue disorder or undifferentiated connective tissue

dysplasia or UCTD).ti,ab,kw,kf. 202870

**15** or/3-4,7-14 470867

**16** (new onset or new presentation$ or newly presenting with or following

or after or post or new diagnosis or newly diagnosed or associated or

link$ with or link$ to or newly developed or new case$ or

association).ti,ab,kw,kf. 14977825

**17** (earlier adj3 infect$).ti,ab,kw,kf. 2277

**18** or/16-17 14978569

**19** 15 and 18 238449

**20** exp severe acute respiratory syndrome coronavirus 2/ or coronavirus

disease 2019/ or experimental coronavirus disease 2019/ 268276

**21** (corona$ adj1 (virus$ or viral$)).ti,ab,kw,kf. 5716

**22** (CoV not (Coefficien$ or co-efficien$ or covalent$ or covington or

covariant$ or covarianc$ or "cut-off value$" or "cutoff value$" or "cutoff

volume$" or "cutoff volume$" or "combined optimi#ation value$" or

"central vessel trunk" or CoVR or CoVS)).ti,ab,kw,kf. 106761

**23** (coronavirus$ or 2019nCoV$ or 19nCoV$ or "2019 novel$" or Ncov$ or

"n-cov" or "SARS-CoV-2$" or "SARSCoV-2$" or SARSCoV2$ or

"SARS-CoV2$" or "severe acute respiratory syndrome$" or

COVID$2).ti,ab,kw,kf. 323220

**24** or/20-23 344489

**25** 19 and 24 2389

**26** exp Adult/ 9958409

27 (adult$ or middle-aged or elderly or men or women or man or

woman).ti,ab,kw,kf. 4540054

**28** or/26-27 11557812

**29** 25 and 28 1763

**30** exp child/ or exp infant/ 2926222

**31** (child$ or adolescen$ or teen$ or preteen$ or paediatric$ or pediatric$

or young people or young person or boy$ or girl$).ti,ab,kw,kf. 2647878

**32** or/30-31 3853618

**33** 25 not 32 2135

**34** 29 or 33 2268

**35** exp animals/ 29012248

**36** humans/ 17545054

**37** 35 not 36 11469392

**38** 34 not 37 2253

**39** limit 38 to english language 2229

**A.3 Cochrane**

Cochrane Central Register of Controlled Trials (CENTRAL)

and Cochrane Database of Systematic Reviews (CDSR)

Results per line

Date: 02/09/2022

**#1** MeSH descriptor: [Autoimmune Diseases] explode all trees 20627

**#2** MeSH descriptor: [Connective Tissue Diseases] explode all trees 10493

**#3** #1 and #2 8043

**#4** ("autoimmune connective tissue" NEXT disease* or "autoimmune

connective tissue" NEXT disorder* or "systemic autoimmune" NEXT

disease$ or "autoimmune CTDs"):TI,AB,KW 85

**#5** (ACTD or ACTDs):TI,AB,KW 21

**#6** "actinomycin D":TI,AB,KW 174

**#7** #5 not #6 7

**#8** MeSH descriptor: [Lupus Erythematosus, Systemic] explode all trees 1193

**#9** MeSH descriptor: [Sjogren's Syndrome] this term only 330

**#10** MeSH descriptor: [Scleroderma, Systemic] explode all trees 628

**#11** MeSH descriptor: [Myositis] explode all trees 223

**#12** MeSH descriptor: [Polymyositis] explode all trees 106

**#13** MeSH descriptor: [Dermatomyositis] this term only 100

**#14** MeSH descriptor: [Mixed Connective Tissue Disease] this term only 4

#15 MeSH descriptor: [Undifferentiated Connective Tissue Diseases] this

term only 9

**#16** (“systemic lupus erythematosus” or SLE or lupus or Sjogren* NEXT

syndrome or Sjoegren* NEXT syndrome or “systemic scleroderma” or

“systemic sclerosis” or inflammatory NEXT myopathy* or “necrotizing

autoimmune myopathy” or myositis or polymyositis or

dermatomyositis or “anti-synthetase syndrome” or “antisynthetase

syndrome” or “mixed connective tissue disease” or “mixed connective

tissue disorder” or “mixed collagen disease” or “undifferentiated

connective tissue disease” or “undifferentiated connective tissue

disorder” or “undifferentiated connective tissue dysplasia” or

UCTD):TI,AB,KW 6853

**#17** {or #3-#4, #7-#16} 13482

**#18** MeSH descriptor: [SARS-CoV-2] this term only 1094

**#19** MeSH descriptor: [COVID-19] this term only 2207

**#20** (corona* NEAR/1 (virus* or viral*)):TI,AB,KW 324

**#21** (CoV not (Coefficien* or co-efficien* or covalent* or Covington* or

covariant* or covarianc* or cut-off NEXT value* or cutoff NEXT value*

or cut-off NEXT volume* or cutoff NEXT volume* or "combined

optimization" NEXT value* or "combined optimisation" NEXT value*

or "central vessel" NEXT trunk* or CoVR or CoVS)):TI,AB,KW 757

**#22** {or #18-#21} 3136

**#23** #17 and #22 16

**A.4 Google Scholar**

Google Scholar Results

Date: 02/09/2022

Search 1:

("autoimmune connective tissue disease" OR "systemic autoimmune

disease" OR "Connective Tissue Disease" OR "systemic lupus

erythematosus" OR lupus OR "Sjogren's Syndrome" OR "systemic

scleroderma") ("COVID-19" OR coronavirus) -child -vaccination

Date Limit: 2019-2022

Search 2:

("mixed connective tissue disease" OR "undifferentiated connective tissue

disease" OR "inflammatory myopathy" OR myositis OR polymyositis OR

dermatomyositis) ("COVID-19" OR coronavirus) -child -vaccination

Date Limit: 2019-2022
